# Supplementary material for: The Construction and Exploration of a Comprehensive MicroRNA Centered Regulatory Network in Foxtail Millet (Setaria italica L.)
Source: Front Plant Sci. 2022 May 6;13:848474. doi: 10.3389/fpls.2022.848474 (PMC9121102; doi:10.3389/fpls.2022.848474)
Supplement: Supplementary file 1 [file Data_Sheet_1.zip › Sit-miR396c_Seita.3G195600.1_607_TPlot.pdf]

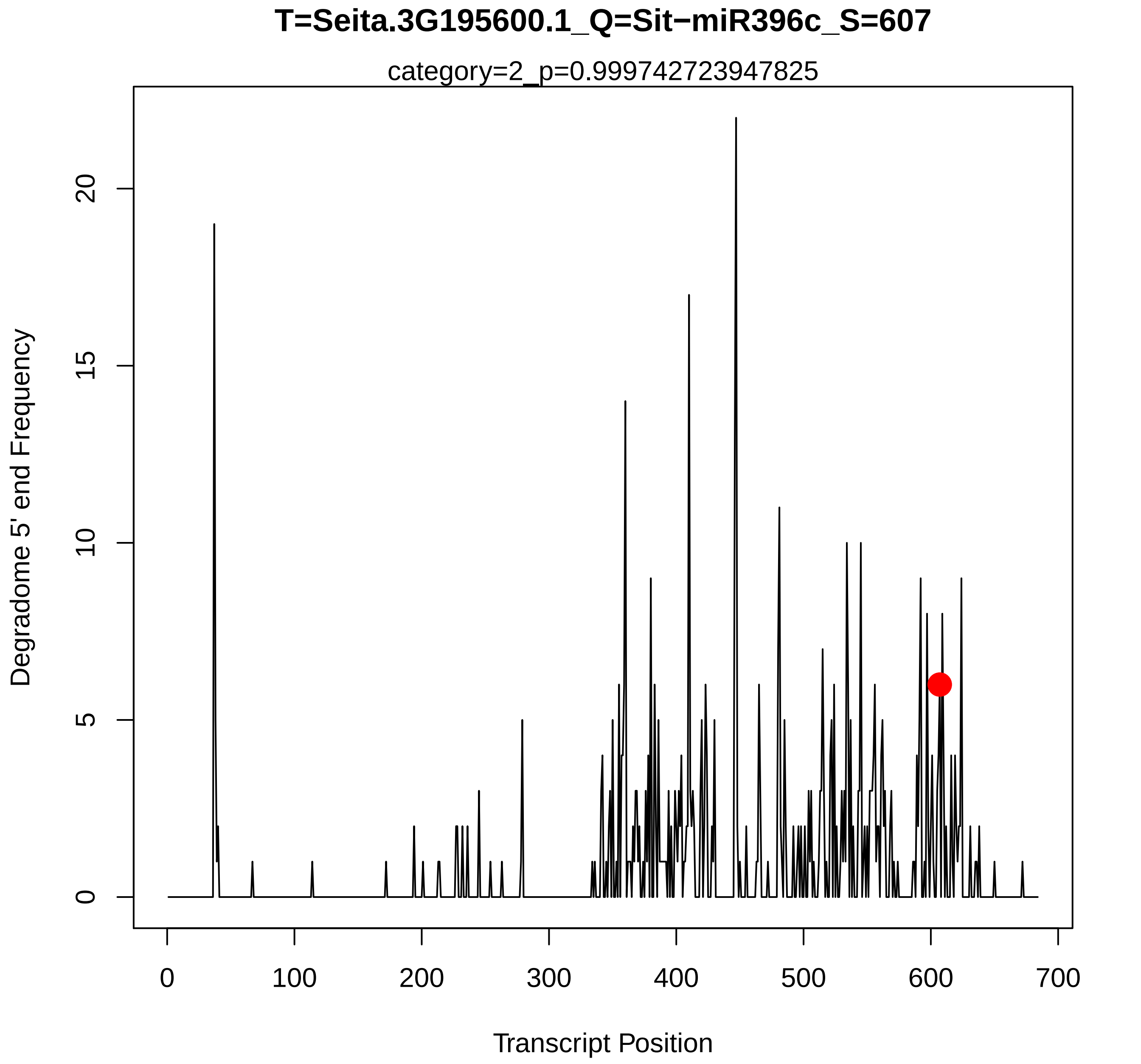

T=Seita.3G195600.1_Q=Sit−miR396c_S=607
category=2_p=0.999742723947825
Degradome 5' end Frequency
Transcript Position
0
5
10
15
20
0
100
200
300
400
500
600
700
